# Structural Phylogenetic Signal Fails at Deep Time: A Bayesian Treebank Analysis of the Transeurasian Languages

**DOI:** 10.3390/e28070816

**Published:** 2026-07-17

**Authors:** Wenchao Li, Haitao Liu

**Affiliations:** 1 School of International Studies, Zhejiang University, Hangzhou 310058, China; widelia@zju.edu.cn; 2 College of Foreign Languages and Literature, Fudan University, Shanghai 200433, China

**Keywords:** phylogenetic signal, Bayesian phylogenetic inference, Shannon entropy, universal dependencies, word order typology, Transeurasian languages, endangered languages

## Abstract

Quantitative phylogenetics in historical linguistics has relied almost entirely on lexical cognate data. This study asks a different question: how much genealogical signal can be recovered from structural features extracted from annotated corpora, and whether it survives at deep time depths. We compute 29 structural features—including Shannon entropies of dependency direction and of dependency-relation distributions, relation-specific directionality ratios, dependency-distance measures, and constructional ratios—across 25 Transeurasian languages from the five proposed groups (Turkic, Mongolic, Tungusic, Japonic, and Koreanic) and three outgroups (Chinese, Vietnamese, and Hindi), 28 languages in all. Most of the Tungusic and Mongolic languages have no running-text corpus, so we built new Universal Dependencies treebanks for them by glossing example sentences from reference grammars; thirteen are used here. Each feature was tested for phylogenetic signal (Pagel’s λ and Blomberg’s K, with FDR correction) under four competing reference topologies, and the features that passed were used for tree inference (Bayesian inference in MrBayes, with Neighbor-Joining as a check). The same pipeline was first run on Indo-European in a companion study, where it recovers only individual subgroups and does not resolve a stable tree. At the depth proposed for the Transeurasian family (a Proto-Transeurasian root of about 9000 years before present), the structural signal was not enough to reconstruct the family’s internal relationships. The signal tests favoured a flat three-way division of the major branches (7 strict/20 relaxed features) over any nested hypothesis (≤2 strict features each), and the strongest signal lay in core word-order parameters (e.g., object direction, λ = 1.00, K = 6.06). But both Bayesian and distance-based inference returned near-complete polytomies: although the chains converged (ASDSF < 0.01), no branch reached a posterior probability above 0.75, and none of the three multi-language branches (Turkic, Mongolic, or Tungusic) was recovered. The outgroup test made the reason clear: Hindi, which is Indo-European but SOV, grouped with the head-final Transeurasian languages rather than with the other two (head-initial) outgroups, so the features are tracking typological similarity, not shared descent, at this depth. The study contributes 13 new treebanks for poorly documented languages, a reproducible framework for testing how much genealogical signal structural features carry, and direct evidence that, at Transeurasian time depths, this signal reflects typology rather than genealogy.

## 1. Introduction

Over the past two decades, the genealogical classification of languages has increasingly drawn on quantitative methods from computational biology. Bayesian phylogenetic inference has produced reconstructions for Indo-European, Austronesian, Sino-Tibetan, Chapacuran, and other families [1,2,3,4,5], building on an older tradition of comparative and lexicostatistical work [6]. Almost all of this work codes its data as binary lexical cognates. Evidence from syntax and morphology, despite its long use in classical comparative linguistics, has played a much smaller part.

There are reasons for this. Whether grammatical structure carries a reliable diachronic signal is itself unsettled. One tradition, going back at least to [7,8], holds that grammatical organization preserves historical information that the lexicon does not, and [9] argues that syntactic features stay relatively stable under moderate contact. A competing tradition, equally old, stresses that structural features are prone to borrowing and parallel innovation [10]. The most recent direct test we know of, ref. [11], uses the SSWL database and finds that syntactic features carry only a weak phylogenetic signal—less than phonological data—and are insufficient to recover deeper, higher-order subgroupings. Structural evidence, in short, is useful but bounded, and the bounds are not well understood.

This is the setting for our approach to the Transeurasian hypothesis [12], which groups Turkic, Mongolic, Tungusic, Japonic, and Koreanic. The hypothesis has been debated for over a century. Supporters and critics disagree not only on whether the five groups share a common ancestor, but on the position of Tungusic, on whether Japonic and Koreanic belong at all, and on how to separate inheritance from the long history of contact across Northeast Asia. Recent lexical-Bayesian work [13] dates the proposed common ancestor to roughly 9000 years before present—far beyond the depth at which structural evidence has so far been shown to be reliable.

Two problems shape this study. The first is data. Treebanks for most Transeurasian languages either do not exist or are too small for this kind of analysis, and the problem is worse for Tungusic and Mongolic, where many languages have no running text at all. We therefore built new Universal Dependencies treebanks for under-documented and endangered Tungusic and Mongolic languages, using a reproducible pipeline that builds UD treebanks from the example sentences of published reference grammars rather than from running text ([14]). Combined with existing treebanks for the remaining Transeurasian languages and three outgroups (Chinese, Vietnamese, and Hindi), this gives a comparative sample of 28 languages, each represented by a single modern variety. The newly built treebanks are scheduled for release in Universal Dependencies (v2.19, November 2026) under the CC BY-SA 4.0 license.

The second problem is time depth. Before applying the pipeline to Transeurasian, we ran it on Indo-European, where the genealogy is known, and a method can be checked against an external answer [15]. The result was clear: of 75 metrics across 16 Indo-European languages, only 4 passed the strict test and 19 the relaxed; trees built on these pinned down individual subgroups (Romance, or Indo-Iranian, depending on the feature set) but did not assemble a stable topology—Germanic, for instance, was not recovered. Read together with [11], the Indo-European results point to a ceiling: structural data yield unstable fragments at shallow depths and, as we show here, nothing at deep ones.

This limits what a structure-only study of Transeurasian can claim. The deepest splits in the proposed family lie well above the depth at which the method recovers signal, even in Indo-European, so we do not treat this paper as a test of whether Transeurasian is a valid family. We treat it as a question about evidence: with a method calibrated on a known family (Indo-European), and the best treebank coverage of these languages now available, how much genealogical signal can structural data recover at this depth, and does it suffice to reconstruct the family? We (1) build new treebanks for thirteen under-resourced languages; (2) document the extraction and inference pipeline; (3) test, under four competing reference topologies, how much phylogenetic signal the structural features carry; and (4) attempt tree inference and report where the signal supports a grouping and where it does not. The structural signal, we find, separates broad typological profiles but does not reconstruct the family’s internal genealogy.

Section 2 reviews previous work on the Transeurasian languages and the questions we respond to. Section 3 describes the methodology: language sample and treebank construction (Section 3.1), preprocessing and missing-data assessment (Section 3.2 and Section 3.3), structural metrics (Section 3.4), phylogenetic signal testing (Section 3.5), and tree inference (Section 3.6). Section 4 presents the results—the signal analysis, the tree inference, and the outgroup test. Section 5 reads them against the Indo-European benchmark and the prior literature on Transeurasian. Section 6 concludes.

## 2. The Transeurasian Language Family Hypothesis

Throughout this paper, we use the term Transeurasian, as introduced by [12], for the proposed family uniting Turkic, Mongolic, Tungusic, Japonic, and Koreanic. The older term Altaic is used for historical references and, where convenient, for the Turkic–Mongolic–Tungusic core (sometimes called Micro-Altaic). Ural-Altaic refers to earlier proposals that additionally included Uralic. We adopt these conventions for clarity; they do not signal commitment to any particular position on the genealogical validity of these groupings.

The hypothesis traces its origins to [16] and was later formalized by [17]. It attracted scholarly attention through proposed cognates and phonological correspondences among Turkic, Mongolic, and Tungusic [18,19,20,21]. The reference [22] was the first to suggest adding Japanese to the Ural-Altaic family. In the mid-twentieth century, the authors of [17] included Korean on the basis of sound correspondences, and [18] further argued that Japanese belonged to Altaic, proposing a lineage from Altaic to Proto-Eastern Altaic, ultimately connecting to Old Japanese and Medieval Korean. One study [23] suggested incorporating Ainu.

Supporters emphasize shared phonological, morphological, and syntactic traits: vowel and consonant harmony (e.g., Turkic vowel harmony, Mongolic advanced/retracted tongue root contrasts), agglutinative morphology marking tense, aspect, voice, and case, and a canonical subject–object–verb (SOV) word order. Vowel harmony in Mongolic, for example, contrasts RTR vowels (/a, ʊ, ɔ/) with ATR vowels (/u, e, o/), and Japanese illustrates agglutination and SOV order:

(1) 子供が先生に本を読ませてもらいました.

Kodomo-ga sensei-ni hon-o yoma-sase-te-morai-mashi-ta

child-NOM teacher-DAT book-ACC read-CAUS-TE-receive-POL-PST

“The child was allowed to read a book by the teacher.”

Critics argue that many of these similarities are better explained by contact and borrowing, treating the group as a Sprachbund rather than a genealogical family [24,25,26]. Some studies [18,27] defended the hypothesis, arguing that the criticisms of Clauson and Doerfer target only lexical correspondences, while the strongest evidence lies in shared verbal morphology. Lexicostatistical work [28] has documented moderate cognate overlap and produced extensive etymological dictionaries, but its methods have been criticized for using lenient phonological and semantic criteria [29,30].

A central unresolved issue is the position of Tungusic relative to the other branches. Four competing hypotheses have been proposed: (1) a polytomy among Tungusic, Japonic–Koreanic, and Mongolic–Turkic (Russian Altaic tradition); (2) Tungusic grouped with Japonic–Koreanic, separate from Mongolic and Turkic [18,31]; (3) Tungusic clustered with Mongolic and Turkic as “Altaic,” distinct from Japonic–Koreanic [26,32,33,34]; and (4) Tungusic branching off earliest within a Mongolic–Tungusic–Turkic clade [35].

The genetic affiliation of Japanese is equally debated. Some scholars support its inclusion in Transeurasian [18,36,37], while others propose alternative affiliations or emphasize heavy contact influences. Ref. [38] proposed affinities with Dravidian, citing shared structural features such as agglutinative morphology, SOV order, and the absence of grammatical gender. Several authors have pointed to Austronesian parallels, with Indonesian often considered the closest match [39,40,41]. The evidence cited includes shared vocabulary, pronouns, reduplication, and interrogative markers—for example, Indonesian *kah* and Japanese *ka*, or Japanese *oya* (親 ‘parent’) and Malay *ayah* (‘father’). Building on this, ref. [42] proposed that Japanese (and possibly Korean) emerged as hybrid languages, suggesting that proto-Transeurasian speakers from northwestern Manchuria migrated south into Liaoning, where they were assimilated by an Austronesian-like farming community, producing proto-Japanese and proto-Korean through fusion. Ref. [43] reach a related but distinct typological conclusion: Koreanic and Japonic initially converged toward an Altaic model and later diverged, forming a distinct “Japanese–Korean” grammatical type. Refs. [44,45] takes a stronger position against the hypothesis, arguing that there is no convincing evidence for shared Proto-Altaic lexical items or regular sound correspondences, and that the observed similarities reflect long-term areal contact, layered borrowing, and convergent typological pressure across Northeast Asia rather than shared inheritance. The Transeurasian debate, taken as a whole, involves three broad positions: genealogical unity (Robbeets and colleagues); hybrid origin with partial inheritance and substantial substrate effects [42]; and areal convergence without genealogical unity (Vovin and colleagues).

The reference [13] presented a large-scale interdisciplinary study combining linguistic, archaeological, and genetic evidence. Using Bayesian phylogenetic inference on basic vocabulary, calibrated with historical and archaeological anchor points, they dated the Proto-Transeurasian root to about 9181 years before present (95% HPD 5595–12,793) and linked the family’s dispersal to the spread of millet farming from the West Liao River region. This study has been contested. Re-examining the same datasets, the authors of [46] argued that the linguistic analysis does not meet the standards of traditional historical-comparative scholarship, that the genetic and archaeological data do not conclusively support a farming-driven dispersal, and that, taken together, none of the three strands provides conclusive evidence for a Transeurasian family. Another study [47] used Levenshtein distances and phylogenetic clustering (MEGA) over more than a hundred languages and dialects from Indo-European, Uralic, Altaic, Koreanic, Japonic, Sino-Tibetan, and Austronesian—with Sinitic dialects, Uto-Aztecan, and Dravidian as outgroups—found no support for either Ural-Altaic or Transeurasian, and could not confirm a traditional Altaic family either. The authors, however, recovered a possible Mongolic–Tungusic genealogical unit. Whichever view one takes, the proposed family lies far deeper in time than the depths at which structural data have been shown to carry reliable phylogenetic signal: the authors of [11] found only weak syntactic signal even within established families, insufficient to recover their deeper subgroupings.

This gap matters here, and it is part of why most phylogenetic work on Transeurasian has stayed lexical: at a depth of around 9000 years, structural features have not been shown to carry a reliable signal on their own. We work from the structural side, anyway, using features extracted from annotated corpora—including the thirteen newly built treebanks introduced in Section 3.1—within a Bayesian framework. The aim is not to settle the Transeurasian debate but to ask, against the Indo-European benchmark, how much genealogical signal a structure-only analysis can recover at this depth, and whether it suffices to reconstruct the family. The posterior probabilities we report should therefore be read as evidence about what the method can and cannot do at this depth, not as a verdict on the family.

## 3. Methodology

### 3.1. Language Sample and Treebank Construction

This study analyzes 25 languages spanning the five branches of the hypothesized Transeurasian family. Turkic (8): Azerbaijani, Kazakh, Kyrgyz, Tatar, Turkish, Uyghur, Uzbek, and Yakut. Mongolic (6): Buriat (Buryat), Daur, Kalmyk, Khalkha, Monguor, and Ordos. Tungusic (9): Even, Evenki, Manchu, Nanai, Negidal, Oroch, Udihe, Ulch, and Xibe. Japonic: Japanese. Koreanic: Korean. Each language is represented by one modern variety only.

We also include three outgroup languages, both to root the trees and to test whether the method can tell Transeurasian languages apart from unrelated ones: Chinese (Sino-Tibetan), Vietnamese (Austroasiatic), and Hindi (Indo-European, Indo-Iranian). This brings the full sample to 28 languages. Three considerations motivated the choice. First, none of the three has ever been assigned to Transeurasian or Altaic under any version of the hypothesis. Second, they differ typologically in a useful way: Chinese and Vietnamese are SVO and broadly head-initial, whereas Hindi is SOV yet indisputably Indo-European. The SOV outgroup is crucial. Because Hindi shares the Transeurasian head-final profile but none of its ancestry, its placement tells us whether the method is tracking shared descent or merely shared word order—a profile any SOV language would show. Third, all three have large, high-quality UD treebanks. One might object that two of the outgroups (Chinese, Vietnamese) are both SVO, and that this shared profile could polarise the distance measures and artificially tighten the Transeurasian cluster. Hindi guards against precisely this: as an SOV outgroup, it is the diagnostic case, and the fact that it falls in among the Transeurasian languages shows that the clustering reflects typology rather than a uniform SVO contrast. A verb-initial or flexible-order outgroup would add a further baseline, and we leave this for future work.

Of the 25 Transeurasian languages, 12 were represented by existing publicly available UD treebanks (UD v2.18): the eight Turkic languages, Buriat (Mongolic), Xibe (Tungusic; UD_Xibe-XDT), Japanese, and Korean. The remaining 13—all Tungusic and Mongolic languages for which no running-text corpus exists—were newly constructed using the GDUD pipeline [14], which extracts and glosses example sentences from published grammatical descriptions and converts them into dependency representations under a shared annotation schema. Of the fifteen treebanks produced by the GDUD pipeline, two (Bonan and Dongxiang) exhibited systematic inconsistencies arising from grammatical-description-to-UD conversion—notably unmapped relation labels and copula constructions inconsistent with UD norms—and were excluded from the present analysis owing to unresolved grammatical-description-to-UD conversion issues; they are being revised for inclusion in the forthcoming GDUD release. The 13 retained GDUD treebanks comprise roughly 5700 tokens in total. Together with the three outgroup treebanks (Chinese, Vietnamese, Hindi), the full sample comprises 28 languages.

For languages with existing public UD treebanks, we used the manually annotated treebank that represents the standard modern variety. Where a language had more than one treebank, we chose the largest manually annotated one and avoided treebanks based on learner data, non-standard varieties, or specialized genres. On this basis, we used UD_Chinese-GSDSimp rather than the Hong Kong (mixed Cantonese) or learner (CFL) Chinese treebanks, and UD_Turkish-IMST, UD_Japanese-GSD, UD_Korean-GSD, UD_Hindi-HDTB, and UD_Vietnamese-VTB for the respective languages. Full construction details for the GDUD treebanks, including the annotation schema and the treatment of language-specific phenomena (morphological derivation, possessive indexing, focus marking, copular constructions, clause chaining, and evidential marking), are given in [14]; the specific treebank and version used for each language are listed in Table 1 and Appendix A.

The existing and newly built treebanks differ considerably in size and in orthography. The existing UD treebanks range from a few hundred to over sixteen thousand sentences, whereas the GDUD treebanks are small (39–113 sentences), since they are drawn from the example sentences of reference grammars. The existing treebanks also use language-specific orthographies (e.g., Cyrillic for Buriat, Chinese characters for Chinese), while the GDUD treebanks use a uniform IPA-based transcription. These differences do not affect the dependency-based measures, which are computed from relation labels and word order and have been shown to be stable across corpus sizes [48,49]; the morphological and lexical/phonological measures we initially considered were excluded for other reasons related to cross-treebank comparability (Section 3.4).

The extraction and inference pipeline described in Section 3.2, Section 3.3, Section 3.4, Section 3.5 and Section 3.6 follows the IE companion study [15], with one difference: the morphological and lexical/phonological measures used there were not comparable across the present treebanks (Section 3.4) and were dropped, so the Transeurasian analysis rests on the dependency-based features alone. Holding the rest of the method constant across a family with known genealogy (Indo-European) and a contested one (Transeurasian) lets us read the Transeurasian results against a benchmark rather than an absolute scale. Table 1 reports treebank statistics for all 28 languages.

### 3.2. Treebank Evaluation

The 12 existing Transeurasian treebanks (15 with the three outgroups included) are published UD resources, and their annotation quality is documented in their original releases. The 13 newly constructed GDUD treebanks were evaluated as described in [14]; the main results are summarized here. Annotation quality was assessed by double annotation of 500 sentences (2959 tokens), which gave agreement rates of 97.4% for part-of-speech tagging (Cohen’s κ = 0.93), 94.5% for unlabeled dependency attachment, 92.4% for labeled dependency attachment, and 91.3% for full morphological feature bundles—all above the 70 threshold suggested by [50]. All 13 treebanks passed the official UD validator with no blocking errors. UD v2 defines 37 universal dependency relation types [51]; the treebanks in our sample attest to between 15 and 34 of them. The lowest counts come from the smallest Tungusic treebanks (Evenki and Oroch, 15 each), which reflect their limited size rather than reduced annotation depth; most treebanks attest 20 to 34 relation types.

The existing and GDUD treebanks differ greatly in size, and the GDUD treebanks in particular are small (39–113 sentences). This raises the question of whether the dependency measures are stable at such sizes. Earlier work indicates that they are: ref. [48] found core dependency structures stable across corpus sizes; ref. [52] found dependency directionality consistent across sentence lengths; and ref. [49] found that genre and sentence length together account for less than 0.3% of the variance in dependency metrics (R^2^ < 0.003). A down-sampling experiment on our own data confirms this directly: when the three largest treebanks are sub-sampled to 40 sentences, the core features (DDir_global, H_dir, H_rel, MDD) deviate from their full-corpus values by less than 3%, and usually by less than 1% (Appendix C). The small GDUD treebanks therefore yield reliable feature estimates.

### 3.3. Preprocessing and Missing-Data Assessment

All treebanks were run through the same preprocessing pipeline as the IE companion study [15]: sentences with malformed CoNLL-U structures or incomplete parses were removed; only universal POS tags and UD v2 dependency relations were retained; punctuation, metadata, and non-linguistic material were excluded; and only sentences forming a single-rooted, connected dependency tree were kept. Language-specific dependency subtypes were merged into their universal base relation so that the relation inventory is comparable across treebanks.

Structural metrics differ in how sensitive they are to corpus size and to the frequency of particular constructions, and a feature may be uncomputable for a language when the relevant relation is unattested or too rare. From the 28 treebanks, we initially extracted 40 dependency-direction features. Features missing in more than 25% of languages were then removed: 16 features, all corresponding to intrinsically rare relations (e.g., clf, expl, dislocated, reparandum), were dropped, and 24 were retained. Missingness was concentrated in the smallest treebanks (Kazakh, 31 sentences; Even and Evenki, 39 sentences each) and in these rare relations, and showed no systematic alignment with branch membership. Together with the six constructional features (Section 3.4) and after collinearity screening, this yields the final set of 29 features used in the phylogenetic analysis **(the complete feature matrix is provided in Appendix B)**.

### 3.4. Structural Metrics

The metric inventory comprises 29 features in two families: (a) dependency-based measures (direction, entropy, and distance) and (b) constructional and frequency-based metrics. We also initially considered a morphological richness measure (moving-average mean size of paradigm, MAMSP) and Swadesh-based lexical/phonological measures. However, MAMSP proved not comparable across treebanks due to differences in word segmentation (agglutinative suffixes are treated as separate tokens in some treebanks but not others), and the available Swadesh transcriptions came from heterogeneous sources with inconsistent conventions and writing systems. Both were therefore excluded, and the analysis focuses on dependency-based features extracted under a uniform UD scheme. Table 2 summarizes the two families; the complete list with formal definitions is provided in Appendix D.

#### 3.4.1. Dependency Direction and Entropy

Dependency direction (DDir), originating in [53] dependency grammar, refers to the linear order of a dependent relative to its head within a syntactic dependency relation [54,55,56,57]. It is one of the most established and cross-linguistically comparable word-order features in syntactic typology [49,56,58,59,60]. For each dependency arc (h, d, r) ∈ E, where h is the head, d the dependent, and r the UD relation type, we define an indicator function that is 1 if pos (h) < pos (d) (head-initial) and 0 otherwise (head-final). The global head-initiality ratio DDir_global is the proportion of head-initial dependencies, and the directional entropy is:

Hdir = −pHI log2pHI − pHFlog2pHF

where p_HI and p_HF are the empirical proportions of head-initial and head-final dependencies. H_dir = 0 corresponds to fully fixed linearization; H_dir = 1 to balanced 50/50 ordering. Relation-specific direction ratios DDir (r) are computed analogously over all instances of each UD relation r. (Dependency relations were collapsed to the 37 universal core relations; language-specific subtypes were merged into their base relation to ensure cross-treebank comparability.)

Alongside H_dir, we compute the dependency-relation distribution entropy H_rel:

$$H_{\mathrm{rel}}=-\sum_{i=1}^{N_{r}}p(r_{i})log2p(r_{i})$$

where *p* (r_i) is the empirical frequency of relation type r_i, and N_r is the number of attested relation types. H_rel captures the breadth of a language’s grammatical inventory. To illustrate, consider the Japanese sentence:

(2) 太郎が先生に手紙を書いた.

Tarō-ga sensei-ni tegami-o kai-ta

Taro-NOM teacher-DAT letter-ACC write-PST

‘Taro wrote a letter to the teacher.’

This sentence contains six non-root dependencies. Three are head-final (Tarō ‘Taro’ → kai ‘write’, nsubj; sensei ‘teacher’ → kai ‘write’, obl; tegami ‘letter’ → kai ‘write’, obj), and three are head-initial (ga → Tarō, case; ni → sensei, case; o → tegami, case). The relation-specific direction ratios computed across an entire treebank, rather than on individual sentences, are what the DDir (r) family captures: in this single sentence, DDir (case) = 1, while DDir (nsubj) = DDir (obl) = DDir (obj) = 0. The dependency-relation distribution is itself uneven (case dominates with 3/6, while nsubj, obl, and obj each contribute 1/6), illustrating the kind of asymmetry that H_rel summarizes at the corpus level (H_rel_ ≈ 1.79 bits for this sentence).

#### 3.4.2. Dependency Distance

Mean dependency distance (MDD) and its variance (VarDL) were computed as in the IE companion study. For each non-root dependency, DD (h, d) = |pos (h) − pos (d)|; MDD is the mean and VarDL is the variance across all dependencies. Both are cross-linguistically stable and robust to corpus size [48,57]. Across the 28 treebanks, MDD ranges from approximately 1.5 to 3.6.

#### 3.4.3. Constructional and Frequency Metrics

Six constructional features capture clausal and valency properties not reducible to directionality: complement-clause ratio (CCompRatio = |E_ccomp|/|E|), adverbial-clause ratio (AdvclRatio = |E_advcl|/|E|), adnominal-clause ratio (AclRatio = |E_acl|/|E|), coordination ratio (CoordRatio = |E_conj|/|E|), a transitivity index (Transitivity = |E_obj|/|VERB tokens|), and a case-marking ratio (CaseRatio = |E_case|/|E|).

#### 3.4.4. Collinearity Screening

Before phylogenetic analysis, we screened for collinearity. DDir_balance was an exact linear transform of DDir_global (r = 1.00) and was removed; the remaining features were retained, provided no pair reached |r| ≥ 0.95 (i.e., near-perfect collinearity). The highest surviving correlation was |r| = 0.92—corresponding to about 85% shared variance—between features whose covariation is genuinely typological (e.g., the alignment of adjectival and nominal modifier order, or the correlation between the mean and variance of dependency distance) rather than a coding artefact. We therefore removed only exact redundancy, retaining pairs whose correlation reflects real linguistic covariation. This yields the final set of 29 features.

### 3.5. Phylogenetic Signal Testing

Following the protocol of the IE companion study ([15])—which serves as our methodological benchmark throughout—each structural feature was first evaluated for phylogenetic signal before being used in tree inference. Signal testing here is not a pre-filter applied for convenience; it is itself part of what we are measuring, since the question of how much structural signal is recoverable at this time depth is one of the paper’s central empirical questions.

A challenge in the Transeurasian case is that the family’s internal structure is itself contested (Section 2). Rather than fixing a single reference topology, we ran the signal tests against four alternative reference topologies, each corresponding to one of the competing classifications discussed in Section 2:

**H1.**  *a polytomy among Tungusic, Japonic–Koreanic, and Mongolic–Turkic;*

**H2.**  *(Tungusic, Japonic–Koreanic) sister to (Mongolic, Turkic);*

**H3.**  *(Tungusic, (Mongolic, Turkic)) as an Altaic core, sister to Japonic–Koreanic;*

**H4.**  *(Turkic, (Mongolic, Tungusic)) as a Mongolic–Tungusic–Turkic clade with Tungusic branching off earliest, sister to Japonic–Koreanic.*

In all four topologies, the internal structure of each branch was left as a polytomy, since the contested question concerns the relationships among the major branches rather than within them; polytomies were randomly resolved and assigned unit branch lengths for the signal computation. For each feature and each reference topology, phylogenetic signal was quantified using two complementary statistics: (a) Pagel’s λ, estimated by maximum likelihood and tested against the null λ = 0 with a likelihood-ratio test; and (b) Blomberg’s K, assessed with a permutation test using 999 random tip-label shuffles. Both were computed with phytools::phylosig [61]. To control for multiple comparisons across the 29 features, *p*-values were adjusted with the Benjamini–Hochberg false discovery rate (FDR) procedure separately for each reference topology. Two feature-selection regimes were applied: (a) a strict regime, retaining features significant under both Pagel’s λ and Blomberg’s K (FDR < 0.05); and (b) a relaxed regime, retaining features significant under either test (FDR < 0.1). Because the four reference topologies share the same major branch identities (Turkic, Mongolic, Tungusic, Japonic, Koreanic, outgroup) and differ only in the relationships among them, comparing the number of features that pass under each topology provides a direct, model-light measure of which classification the structural data are most consistent with. Tree inference (Section 3.6) was then carried out on the signal-selected feature sets.

### 3.6. Phylogenetic Inference

The signal-selected features (Section 3.5) were used for tree inference under two configurations: a strict matrix (the 7 features significant under both signal tests at FDR < 0.05) and a relaxed matrix (the 20 features significant under either test at FDR < 0.1). Each continuous feature was discretized into four ordered states by equal-frequency binning (B = 4), so that the resulting multistate characters preserve the rank order of the original measure while being usable with Mk-type substitution models [62]. Missing values, which were few (Section 3.3), were assigned the column mean before binning.

Bayesian inference was performed in MrBayes 3.2.7 [63] under a standard discrete model with variable coding and gamma-distributed rate variation across characters (lset coding = variable rates = gamma), with a symmetric Dirichlet prior on state frequencies. For each matrix, four independent runs of four chains each were run for 2,000,000 generations, sampling every 500 generations, with the first 25% discarded as burn-in. Convergence was assessed by the average standard deviation of split frequencies (ASDSF), with a target below 0.01. Both analyses reached this target (ASDSF = 0.0083 for the strict matrix and 0.0094 for the relaxed matrix). Results were summarized as 50% majority-rule consensus trees with posterior probabilities (PP) on the resolved nodes.

The tree was rooted in Chinese through the MrBayes outgroup command. Chinese was chosen because it is head-initial and clearly lies outside the head-final Transeurasian profile; since the three outgroups do not form a clade (Section 4.3), a single typologically distinct outgroup yields a cleaner root than the three combined. Because the ingroup is itself essentially unresolved, the position of the root has little bearing on the (polytomous) topology. No topological constraints were imposed within the sample, so the data alone determined the grouping of the 25 Transeurasian languages. The placement of the outgroups provides a check on what the method is tracking: if the inference reflects genealogy, the three genealogically unrelated outgroups should fall outside the Transeurasian sample, and Hindi in particular—Indo-European but, like the Transeurasian languages, SOV and head-final—should not be drawn in among them. If instead Hindi groups with the head-final Transeurasian languages, this would indicate that the features respond to typological similarity rather than to shared descent (see results in Section 4.3).

As a methodological check, the same signal-selected matrices were also analyzed with distance-based Neighbor-Joining [64], using Euclidean distances on the z-scored features, with node support from 1000 bootstrap replicates. To confirm that the four-bin discretization does not drive the result, we repeated the Bayesian analysis with B = 3 and B = 5 (all chains converged, ASDSF ≤ 0.010). The outcome was unchanged: the strict matrices returned near-complete or fully star-like polytomies (B = 3 strict resolved no internal clade at all), and no analysis recovered any of the major branches. The few resolved pairs varied with B and were typologically or genealogically incoherent—(Nanai, Vietnamese) at B = 3 and (Yakut, Kalmyk) at B = 5, each crossing established branch boundaries or joining an outgroup—consistent with noise rather than signal. The unresolved topology is therefore not an artefact of the binning threshold. We note that equal-frequency binning yields ordered states, whereas the standard Mk model assumes symmetric, unordered rates; we treat this as a conservative simplification.

## 4. Results

We report the results in three steps. Section 4.1 presents the phylogenetic-signal analysis; Section 4.2 presents the tree inference (Bayesian and NJ); Section 4.3 examines the outgroup and the typological control (Hindi). Throughout, we read the results against the Indo-European benchmark [15] and against [11], finding that syntactic data carry only weak phylogenetic signal at depth.

### 4.1. Phylogenetic Signal Across Reference Topologies

Each of the 29 features was tested for phylogenetic signal under the four reference topologies (H1–H4) defined in Section 3.5, using Pagel’s λ and Blomberg’s K with Benjamini–Hochberg FDR correction. The number of features passing the signal tests differed sharply across topologies (Table 3).

Under the three-way polytomy (H1: Turkic–Mongolic, Tungusic, and Japonic–Koreanic as parallel branches), 7 features passed the strict criterion (both λ and K significant at FDR < 0.05), and 20 passed the relaxed criterion (either test at FDR < 0.1). The three nested hypotheses yielded a much weaker signal: under H2, H3, and H4, only 1, 2, and 0 features passed the strict criterion, and 3, 6, and 16 the relaxed criterion, respectively. This comparison is a heuristic rather than a formal model test, and H1, being the least resolved reference (a trichotomy), is also the easiest for features to be consistent with; the counts are best read as showing no support for any specific nested pairing rather than as positive evidence for a flat division. (The relaxed counts are, moreover, threshold-dependent: H4 passes are nonstrict but 16 relaxed.) We also note that the features used for inference were selected under H1, which is a mild and conservative form of circularity. Read cautiously, the pattern indicates that the structural data do not favor any particular internal nesting of the major branches. This already anticipates the tree-inference results below: the data carry enough signal to separate broad typological groupings but not to resolve their internal branching.

The features carrying the strongest signal were all core word-order properties. Under H1, the seven strict features were the directionality of objects (DDir_obj; λ = 1.00, K = 6.06), oblique arguments (DDir_obl; λ = 1.00, K = 2.09), open complements (DDir_xcomp; λ = 1.00, K = 2.69), case marking (DDir_case; λ = 1.00, K = 1.83), relative/adnominal clauses (DDir_acl; λ = 1.00, K = 1.20), complement clauses (DDir_ccomp; λ = 0.89, K = 0.99), and the global head-initiality ratio (DDir_global; λ = 0.96, K = 2.01) (Table 4). Values of λ at or near 1 and K above 1 indicate that these features are distributed across languages in close accordance with—or more conservatively than—a Brownian-motion model of descent. That the signal concentrates in the object–verb and head-direction parameters is expected: these are among the most diachronically stable word-order properties and most clearly separate the consistently head-final Transeurasian languages from the head-initial outgroups (Chinese, Vietnamese).

### 4.2. Tree Inference

We inferred trees from both the strict (7-feature) and relaxed (20-feature) signal-selected matrices, using Bayesian inference (MrBayes) and, as a check, distance-based Neighbor-Joining (NJ).

**Figure 1:**
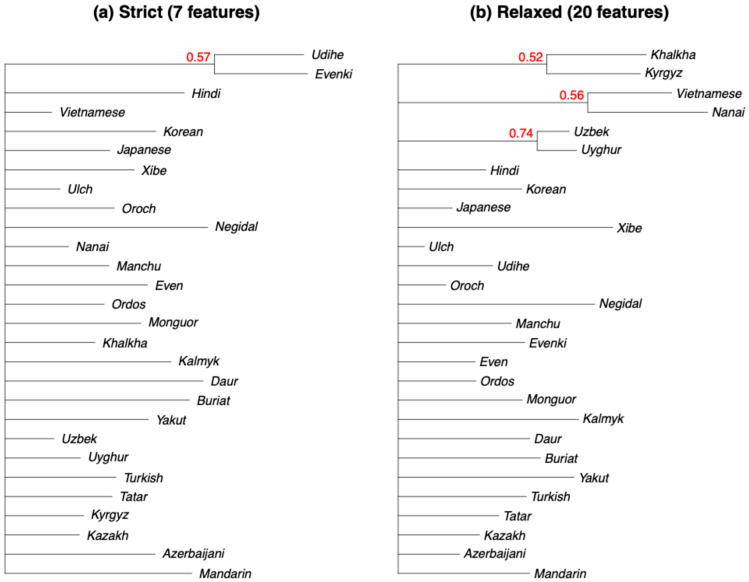
Bayesian majority-rule consensus trees from the strict (7-feature) and relaxed (20-feature) matrices. Both are near-complete polytomies; posterior probabilities are shown on the few resolved nodes.

The Bayesian analyses converged in both cases (average standard deviation of split frequencies, ASDSF = 0.0083 for strict and 0.0094 for relaxed, both below the 0.01 target). Despite convergence, the majority-rule consensus trees were almost completely unresolved (Figure 1). Under the strict matrix, only a single internal clade was recovered—(Evenki, Udihe)—with a posterior probability (PP) of 0.57. Under the relaxed matrix, only three shallow pairs were resolved—(Uyghur, Uzbek) at PP = 0.74, (Nanai, Vietnamese) at PP = 0.56, and (Kyrgyz, Khalkha) at PP = 0.52. No branch reached PP > 0.75, and none of the three multi-language branches (Turkic, Mongolic, Tungusic) was recovered as a clade (Japonic and Koreanic are represented by single languages, so monophyly is not testable for them). That the chains converged yet returned a near-complete polytomy is itself informative: it indicates not a failure of sampling but an absence of recoverable internal signal in the data.

The seven strict features are all head-directionality ratios and share a common dominant dimension: their pairwise Pearson correlations average |r| = 0.53 (range 0.17–0.86), and their first principal component accounts for 60% of the variance, with all directionality features loading in the same direction and case marking in the opposite one (as expected, since case markers are post-nominal in head-final languages). They are therefore not seven independent characters but largely reflect a single head-finality axis, while the Mk model treats them as independent. If anything, this near-collinearity should inflate apparent resolution through pseudo-replication; that inference, nonetheless, yields a polytomy, indicating the unresolved outcome is conservative.

The distance-based NJ trees told the same story (Appendix E). They did not recover any of the three multi-language branches (Turkic, Mongolic, Tungusic) as monophyletic either, and the few groupings that did appear were typologically rather than genealogically coherent: the head-final languages (Turkic, Mongolic, Tungusic, Japonic, and Koreanic) clustered loosely together, while the head-initial outgroups, Chinese and Vietnamese, were placed on long, separate branches. The two inference methods thus agree that the structural data cannot reconstruct Transeurasian internal relationships at this time depth.

### 4.3. Outgroup Behaviour and the Typological Control

The three outgroups did not behave as a clean genealogical outgroup, and this is revealing rather than problematic. In the NJ analysis, the three outgroups did not form a single clade outside the Transeurasian sample: Chinese and Vietnamese (SVO, head-initial) were placed apart on long branches, whereas Hindi—although Indo-European, and therefore genealogically unrelated to the Transeurasian languages—fell among the head-final Transeurasian languages rather than with the other two outgroups.

This is exactly the diagnostic for which the inclusion of an SOV outgroup was designed (Section 3.1). Hindi shares SOV/head-final order with the Transeurasian languages but shows no genealogical relationship with them; its placement among them shows that the structural features respond, at this depth, to typological similarity (head-directionality) rather than to shared descent. The same point is evident internally: the only “clades” recovered cut across established branches (e.g., Nanai with Vietnamese, Kyrgyz with Khalkha), consistent with similarity-based rather than genealogy-based clustering.

The three analyses converge on a single conclusion. At the time depth proposed for the Transeurasian family (a Proto-Transeurasian root of about 9000 years before present; [13]), structural features do not carry enough phylogenetic signal to reconstruct the internal relationships of the family. They distinguish broad head-final from head-initial profiles, but the resulting groupings reflect typology rather than genealogy. This is consistent with the Indo-European benchmark [15], where the same pipeline recovers only isolated, unstable subgroups rather than a coherent tree, and with the authors of [11] finding that syntactic data carry only weak phylogenetic signal, insufficient to recover deeper subgroupings.

## 5. Discussion

This study did not set out to adjudicate the Transeurasian hypothesis but to test whether structural phylogenetic signal survives at deep time depths, using Transeurasian as a particularly difficult test case. The results give a clear, if negative, answer. At the time depth proposed for the family—around 9000 years before present for the Proto-Transeurasian root [13]—structural features extracted from dependency treebanks do not carry enough signal to reconstruct internal relationships: both Bayesian and distance-based inference returned near-complete polytomies, no clade reached a posterior probability above 0.75, and none of the three testable branches (Turkic, Mongolic, Tungusic) formed a clade.

### 5.1. Signal Without Resolution

The most informative result is the gap between the signal tests and the tree inference. The signal tests were not empty. Seven features passed the strict criterion, and the strongest of them—object direction, oblique direction, and the global head-initiality ratio—had λ values at or near 1 and K values well above 1, which normally indicates strong phylogenetic structure. But these same features did not resolve a tree. This gap is the main finding. What λ and K measure is whether a feature’s values line up with a given tree—not whether the feature can tell competing trees apart. The features that “pass” here do so because they all track the same thing: the deepest typological split in the sample, between head-final and head-initial order. That split matches the broadest grouping (the three-way division in H1), but it says nothing about how the branches relate below that level. Therefore, the signal is real, but it is coarse. It tells head-final languages apart from head-initial ones; it does not recover lineages. This is why the signal tests favour the flat three-way topology (H1: 7 strict/20 relaxed) over every nested hypothesis (H2–H4: ≤2 strict), and why the Bayesian consensus is itself a near-polytomy. The two analyses point to the same thing: the data support a broad three-way division but cannot resolve the relationships within it. The result also has an information-theoretic reading suited to the present context. Of our two Shannon-entropy features, neither carries phylogenetic signal, whereas the directional-bias ratios do. This asymmetry is interpretable: the relation-distribution entropy H_rel measures how evenly a language spreads its dependency relations—a property shaped by discourse, genre, and processing pressures shared across languages—so it converges on similar values regardless of ancestry and carries little historical information. Head-directionality, by contrast, is a low-entropy, near-categorical parameter (most languages are consistently head-final or head-initial), and it is precisely this near-determinacy that lets it separate broad typological classes. At this depth, then, what little historical information survives resides in the categorical word-order bias, not in the distributional entropy of the grammar.

### 5.2. Typology, Not Genealogy

The outgroup behavior makes this clear. Hindi was included because it is Indo-European—genealogically unrelated to any Transeurasian language—but SOV and head-final, like them. Had the method been tracking descent, Hindi should have grouped with the other outgroups (Chinese, Vietnamese) outside the Transeurasian sample. Instead, it fell among the head-final Transeurasian languages. The few groupings that the trees did recover point the same way: pairs such as Nanai with Vietnamese, or Kyrgyz with Khalkha, cut across established branches and are coherent only as similarity, not as inheritance.

The conclusion is therefore not that the Transeurasian languages are unrelated, nor that they are related—the structural data are simply mute on that question at this depth. What they show is narrower: dependency-based structural features index typological profile rather than genealogy. The clustering of Turkic, Mongolic, Tungusic, Japonic, and Koreanic that one does see is just what we would expect if their shared SOV, head-final, agglutinative profile arose, or was maintained, through prolonged areal contact and convergence across Northeast Asia—the position long argued by [44] and by the Sprachbund tradition [24,25]. The mechanisms underlying such convergences are well documented in contact linguistics. Prolonged, intense contact of the kind posited for Northeast Asia can spread head-final, agglutinative structure through pattern replication and grammatical calquing—the copying of syntactic templates without the copying of forms—and through the reanalysis of borrowed material along native lines. Crucially, these processes produce a shared structure that is hierarchically distributed across languages in a way that can statistically mimic the nested pattern expected from common descent, which is exactly why structural similarity alone cannot arbitrate between inheritance and contact at this depth.

Head-directionality is, in any case, a coarse, low-resolution feature: SOV order is found in many unrelated languages worldwide, including those with no possibility of mutual contact, so it cannot, on its own, distinguish inheritance from convergence. Our results do not prove that account, but they are fully compatible with it, and show that structural similarity among these languages cannot by itself be taken as evidence of common descent.

### 5.3. A Data Limit, Not a Method Failure

A negative result is different from a flawed one, and three factors show that the lack of resolution stems from the data at this depth rather than from the method. First, the same pipeline applied to Indo-European in the companion study [15] does find structure where structure exists, though even there the signal is limited: it pins down individual subgroups—Romance (PP = 0.79) or Indo-Iranian (PP = 0.999), depending on the feature set—while failing to assemble them into a stable tree. That the method recovers well-differentiated shallow clades in a family of known genealogy, from matrices as small as four to nineteen characters, shows it can extract signal where signal exists; that it recovers none at Transeurasian depths places the difference at the level of time depth, not method or matrix size (in the Indo-European study the signal was itself unstable—the two feature sets yielded largely different topologies, and Germanic was not recovered—so the contrast is not IE-success versus Transeurasian-failure, but unstable fragments at shallower depth giving way to none at greater depth). Second, the Bayesian chains converged (ASDSF < 0.01) but still returned a polytomy; the lack of resolution therefore reflects the data rather than undersampling. Third, two different methods (Bayesian and NJ) and two feature sets (strict and relaxed) gave the same outcome. A down-sampling experiment (Appendix C) further rules out corpus size as a cause: core features computed from sub-samples of 40 sentences deviate from their full-corpus values by under 3%, so the small treebanks are not the source of the non-resolution. The result also lines up with external evidence. The loss of resolution is consistent with the authors’ more general finding [11] that syntactic data carry only weak phylogenetic signal and do not recover deeper, higher-order subgroupings. It converges, too, with work on the lexical side: the authors of [47], using Levenshtein distances and phylogenetic clustering rather than structural features, likewise found no support for Ural-Altaic or Transeurasian. That two independent lines of quantitative evidence—lexical distance and dependency structure—both fail to recover deep Transeurasian unity is harder to dismiss than either result alone. The two studies do differ on one point: Ref. [47] recover a Mongolic–Tungusic grouping on lexical grounds, whereas our structural data resolve no such clade. This is not a contradiction; it follows directly from our central finding that the structural signal is too weak at this depth to resolve any internal grouping, and so says nothing for or against the Mongolic–Tungusic unit, which remains a question for lexical and other evidence.

### 5.4. Contributions and Limitations

Beyond the negative phylogenetic result, the study makes two positive contributions. First, it releases new Universal Dependencies treebanks for under-resourced and endangered Tungusic and Mongolic languages (thirteen used here, with two further treebanks to be added once revised), built from reference grammars with a reproducible pipeline; these are usable for parsing, typological comparison, and language documentation independently of the present analysis. Second, it offers a transferable framework for asking not whether structural data support a given tree, but how much genealogical signal they carry—calibrated on a known family (Indo-European) and applied to a contested one.

Our findings also speak to a broader, ongoing scrutiny of phylogenetic methods in linguistics. d’Huy [65], comparing three recent attempts to build a global language tree—from structural typology, phoneticized lexicon, and mixed Bayesian models—questions the validity of the tree model at great time depths, yet notes that some deep clusterings do appear to survive the noise of contact and convergence. Our results sharpen one side of this picture: for dependency-based structural features at Transeurasian depths, the noise overwhelms the genealogical signal altogether. Whether a more robust deep structure can be recovered from other kinds of data, as the synthesis by [65] suggests, is a question our study cannot settle, which points to its main limitation.

The analysis is restricted to dependency-based structural features. Morphological richness and lexical/phonological measures were excluded because they were not comparable across treebanks (Section 3.4), and a fuller picture might combine structural with lexical-cognate evidence. The exclusion of the morphological-richness measure also points to a broader obstacle for quantitative typology of agglutinative languages: whether an agglutinative suffix is treated as a morphological feature on its host or as a separate syntactic token drastically changes token counts, dependency distances, and relation-distribution entropy. Until segmentation is harmonized across treebanks, morphological and distance-based comparisons of agglutinative languages will remain sensitive to this annotation choice. The treebanks also vary greatly in size and provenance, and although the core features proved robust to corpus size (Appendix C), the smallest treebanks inevitably attest fewer relation types. A further limitation concerns the register. The thirteen GDUD treebanks are built from the illustrative example sentences of reference grammars, whereas the existing treebanks draw on news, fiction, and other running text. Grammarians’ examples are not a representative sample: they are chosen to display particular constructions and may over-represent marked patterns and under-represent the complex subordination and discourse-driven variation of naturalistic text. This matters little for the head-directionality ratios that carry the signal here—word order in a transitive clause is stable whatever its source—but it could affect the frequency-based features (H_rel, the constructional ratios, and the rarer relation-specific ratios). Our down-sampling check (Appendix C) addresses corpus size but not composition; the frequency-based conclusions should therefore be read with this caveat, and the core findings rest on the directionality features, which are the least register-sensitive. The analysis is also confined to one modern variety per language. Where earlier stages can be treebanked—Old Turkic, Middle Mongol [32], Old Japanese—including them would shorten the unobserved branches back toward the proposed common ancestor and could sharpen a deep-time test; this is a natural extension we leave for future work. Finally, our conclusion concerns what structural data can and cannot do at this time depth—not the validity of the Transeurasian hypothesis itself. The structural evidence is silent on whether these languages share a common ancestor; lexical, archaeological, and genetic lines of evidence [13] bear on that question, but this study does not address them.

## 6. Conclusions

Structural features from dependency treebanks can be tested for the amount of genealogical signal they carry. We ran such a test on the Transeurasian languages, using 29 features across 28 languages and a pipeline first calibrated on Indo-European. If the family is valid, its internal divergences reach back some 9000 years [13]—far deeper than the depth at which structural signal has been shown to be reliable. At that depth, the signal was enough to separate broad typological profiles but not to reconstruct the family’s internal relationships. Both Bayesian and distance-based inference returned near-complete polytomies; no branch reached a posterior probability above 0.75; and an Indo-European control language, Hindi—included precisely because it is SOV but unrelated—was pulled into the head-final Transeurasian cluster. Structural similarity, at this depth, reflects typology, not descent.

This is a limit of the data at this depth, not of the method: the same pipeline recovers individual subgroups in Indo-European (though even there the tree is unstable), the Bayesian chains converged, and two inference methods and two feature sets agreed. The negative result is therefore robust, and it is consistent with the weak deep-level signal that [11] report for syntactic data more generally. Beyond this finding, the study leaves behind two things: new UD treebanks for under-resourced and endangered Tungusic and Mongolic languages, built from reference grammars, and a reproducible way to test how much genealogical signal structural features carry, calibrated on a known family and applied to a contested one. Whether the Transeurasian languages ultimately share a common ancestor is a question for lexical, archaeological, and genetic evidence. What this study shows is what structural data, on their own, can and cannot do at this time depth.

## Figures and Tables

**Table 1:** Treebank statistics for all 28 languages.

Branch	Language	Source	Sentence	Tokens	DepLinks
Turkic	Azerbaijani	UD_Azerbaijani-TueCL	109	663	420
Turkic	Kazakh	UD_Kazakh-KTB	31	529	384
Turkic	Kyrgyz	UD_Kyrgyz-KTMU	1258	11,883	8927
Turkic	Tatar	UD_Tatar-NMCTT	148	2280	1764
Turkic	Turkish	UD_Turkish-IMST	5635	58,096	42,052
Turkic	Uyghur	UD_Uyghur-UDT	1656	19,262	13,906
Turkic	Uzbek	UD_Uzbek-UT	500	5850	4530
Turkic	Yakut	UD_Yakut-YKTDT	299	1460	1012
Mongolic	Buriat	UD_Buryat-BDT	908	10,032	7309
Mongolic	Daur	self-built (GDUD)	95	659	455
Mongolic	Kalmyk	self-built (GDUD)	92	483	332
Mongolic	Khalkha	self-built (GDUD)	88	473	323
Mongolic	Monguor (Tu)	self-built (GDUD)	77	593	445
Mongolic	Ordos	self-built (GDUD)	85	293	193
Tungusic	Xibe	UD_Xibe-XDT	810	15,401	12,696
Tungusic	Even	self-built (GDUD)	39	282	224
Tungusic	Evenki	self-built (GDUD)	39	249	167
Tungusic	Manchu	self-built (GDUD)	82	445	362
Tungusic	Nanai	self-built (GDUD)	113	821	709
Tungusic	Negidal	self-built (GDUD)	62	281	205
Tungusic	Oroch	self-built (GDUD)	110	347	155
Tungusic	Udihe	self-built (GDUD)	94	504	333
Tungusic	Ulch	self-built (GDUD)	93	232	131
Japonic	Japanese	UD_Japanese-GSD	8100	193,654	166,321
Koreanic	Korean	UD_Korean-GSD	6339	80,322	63,572
Outgroup	Chinese	UD_Chinese-GSDSimp	4997	123,289	101,227
Outgroup	Vietnamese	UD_Vietnamese-VTB	3323	58,069	46,595
Outgroup	Hindi	UD_Hindi-HDTB	16,649	351,704	311,600

**Table 2:** The two metric families and their features.

	Features	Examples
(a) Dependency-based	23	DDir_global, H_dir, H_rel, MDD, VarDL, and relation-specific DDir(r) for acl, advcl, advmod, amod, case, cc, ccomp, compound, conj, det, discourse, nmod, nsubj, nummod, obj, obl, parataxis, xcomp
(b) Constructional/frequency	6	CCompRatio, AdvclRatio, AclRatio, CoordRatio, Transitivity, CaseRatio

**Table 3:** Number of features passing the signal tests under each reference topology.

Reference Topology	Strict (Both λ and K, FDR < 0.05)	Relaxed (Either Test, FDR < 0.1)
**H1.** *three-way polytomy (TM/Tg/JK)*	7	20
**H2.** *(Tungusic, Japonic–Koreanic) vs. (Mongolic, Turkic)*	1	3
**H3.** *(Tungusic, (Mongolic, Turkic)) vs. Japonic–Koreanic*	2	6
**H4.** *(Turkic, (Mongolic, Tungusic)) vs. Japonic–Koreanic*	0	16

TM—Turkic–Mongolic; Tg—Tungusic; JK—Japonic–Koreanic. Strict—features significant under both Pagel’s λ and Blomberg’s K (FDR < 0.05); Relaxed—features significant under either test (FDR < 0.1). Out of 29 features tested.

**Table 4:** Strict signal-bearing features under reference topology H1 (significant under both Pagel’s λ and Blomberg’s K at FDR < 0.05), ordered by Blomberg’s K.

Feature	Relation	λ	λ (FDR)	K	K (FDR)
DDir_obj	direction of objects	1.00	<0.001	6.06	0.015
DDir_xcomp	direction of open complements	1.00	<0.001	2.69	0.019
DDir_obl	direction of oblique arguments	1.00	<0.001	2.09	0.044
DDir_global	global head-initiality ratio	0.96	<0.001	2.01	0.015
DDir_case	direction of case marking	1.00	<0.001	1.83	0.044
DDir_acl	direction of adnominal clauses	1.00	<0.001	1.20	0.050
DDir_ccomp	direction of complement clauses	0.89	0.005	0.99	0.044

λ—Pagel’s lambda; K—Blomberg’s K. Both statistics were computed with phytools:phylosig and corrected for multiple comparisons (Benjamini–Hochberg FDR) across the 29 features. Several λ estimates lie at the upper boundary (λ = 1.00), where maximum-likelihood values commonly pin; the likelihood-ratio test against λ = 0 remains valid, but the point estimate itself should not be over-interpreted. A value of K > 1 means that trait values are more similar within groups (more clumped on the reference tree) than expected under Brownian motion, i.e., strong phylogenetic structure.

**Figure A1:**
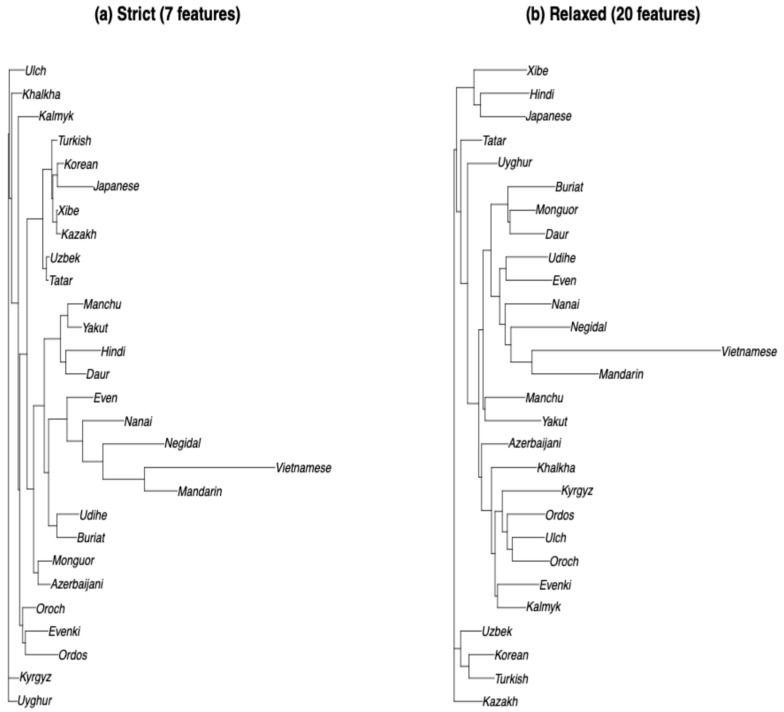
Neighbor-Joining trees from the strict (7-feature, left) and relaxed (20-feature, right) signal-selected matrices, using Euclidean distances on z-scored features. Neither tree recovers any of the three multi-language branches (Turkic, Mongolic, Tungusic) as coherent groups; the head-final languages cluster loosely together while the head-initial outgroups (Chinese, Vietnamese) fall on long, separate branches, and Hindi—although Indo-European—groups among the head-final Transeurasian languages. The recovered groupings are typological rather than genealogical, consistent with the near-complete polytomies obtained under Bayesian inference (Figure 1).

## Data Availability

The 15 existing UD treebanks used here (12 Transeurasian and three outgroups) are publicly available from the Universal Dependencies project (https://universaldependencies.org (accessed on 14 July 2026)): the eight Turkic treebanks (Azerbaijani-TueCL, Kazakh-KTB, Kyrgyz-KTMU, Tatar-NMCTT, Turkish-IMST, Uyghur-UDT, Uzbek-UT, Yakut-YKTDT), Buryat-BDT, Xibe-XDT, Japanese-GSD, Korean-GSD, and the three outgroups (Chinese-GSDSimp, Vietnamese-VTB, and Hindi-HDTB). The 13 treebanks newly constructed for this study (Daur, Kalmyk, Khalkha, Monguor, Ordos, Even, Evenki, Manchu, Nanai, Negidal, Oroch, Udihe, and Ulch) are scheduled for release through Universal Dependencies (version 2.19, November 2026) under the CC BY-SA 4.0 license; until then, they are available from the corresponding author on request. All structural metrics were computed with custom scripts; the feature matrices, signal-test results, and tree files are provided in the Supplementary Materials and are available at https://github.com/wenchao-li-zju/transeurasian-structural-signal (accessed on 14 July 2026).

## Supplementary Materials

The following supporting information can be downloaded at: https://www.mdpi.com/article/10.3390/e28070816/s1.

## Appendix A. Data Sources and Treebank Statistics

**#**	**Branch**	**Language**	**Treebank/Source**	**Text Type**	**Sentences**	**Tokens**
1	Turkic	Azerbaijani	UD_Azerbaijani-TueCL	Grammar examples	109	663
2	Turkic	Kazakh	UD_Kazakh-KTB	News, fiction, wiki	31	529
3	Turkic	Kyrgyz	UD_Kyrgyz-KTMU	Grammar examples	1258	11,883
4	Turkic	Tatar	UD_Tatar-NMCTT	Nonfiction, news	148	2280
5	Turkic	Turkish	UD_Turkish-IMST	Nonfiction, news	5635	58,096
6	Turkic	Uyghur	UD_Uyghur-UDT	Fiction	1656	19,262
7	Turkic	Uzbek	UD_Uzbek-UT	News, fiction	500	5850
8	Turkic	Yakut	UD_Yakut-YKTDT	Nonfiction, news	299	1460
9	Mongolic	Buriat	UD_Buryat-BDT	Grammar examples, news, fiction	908	10,032
10	Mongolic	Daur	Self-built (GDUD)	Grammar examples	95	659
11	Mongolic	Kalmyk	Self-built (GDUD)	Grammar examples	92	483
12	Mongolic	Khalkha	Self-built (GDUD)	Grammar examples	88	473
13	Mongolic	Monguor (Tu)	Self-built (GDUD)	Grammar examples	77	593
14	Mongolic	Ordos	Self-built (GDUD)	Grammar examples	85	293
15	Tungusic	Even	Self-built (GDUD)	Grammar examples	39	282
16	Tungusic	Evenki	Self-built (GDUD)	Grammar examples	39	249
17	Tungusic	Manchu	Self-built (GDUD)	Grammar examples	82	445
18	Tungusic	Nanai	Self-built (GDUD)	Grammar examples	113	821
19	Tungusic	Negidal	Self-built (GDUD)	Grammar examples	62	281
20	Tungusic	Oroch	Self-built (GDUD)	Grammar examples	110	347
21	Tungusic	Udihe	Self-built (GDUD)	Grammar examples	94	504
22	Tungusic	Ulch	Self-built (GDUD)	Grammar examples	93	232
23	Tungusic	Xibe	UD_Xibe-XDT	Grammar examples, newspaper, textbooks	810	15,401
24	Japonic	Japanese	UD_Japanese-GSD	news, blog	8100	193,654
25	Koreanic	Korean	UD_Korean-GSD	news, blog	6339	80,322
26	Outgroup	Chinese	UD_Chinese-GSDSimp	Wiki	4997	123,289
27	Outgroup	Vietnamese	UD_Vietnamese-VTB	News	3323	58,069
28	Outgroup	Hindi	UD_Hindi-HDTB	News	16,649	351,704

## Appendix B. Feature Matrix

The complete 28 × 29 matrix of structural feature values is available in the GitHub (version 3.6.2) repository (Data Availability) as features_final_29.csv.

## Appendix C. Robustness of Structural Features to Corpus Size

A potential concern is that the smallest treebanks in our sample (e.g., Kazakh, 31 sentences; Even and Evenki, 39 sentences each) are too small to yield reliable feature values. To assess this, we conducted a down-sampling experiment on three of the largest treebanks—Turkish (5635 sentences), Japanese (8100), and Hindi (16649). For each, we randomly drew sub-samples of 40 and 90 sentences (approximating the size of the smallest GDUD treebanks), repeated 20 times, and recomputed four core features (DDir_global, H_dir, H_rel, and MDD). We then compared the mean of the sub-sampled values to the full-corpus value.

The features proved highly stable. At a sub-sample size of 40 sentences, the mean sub-sampled values deviated from the full-corpus values by at most 2.1% (DDir_global), and typically by less than 1% (Table A1). At 90 sentences, deviations were below 1% for nearly all features. Standard deviations across the 20 repetitions were likewise small (e.g., H_dir ± 0.002–0.035). These results indicate that the core dependency-based features are recoverable from samples as small as 40 sentences, supporting the inclusion of the smaller GDUD treebanks in the analysis.

**Table A1:** Down-sampling stability (mean ± SD over 20 repetitions; deviation from full-corpus value in parentheses).

Treebank	Feature	Full	n = 40	n = 90
Turkish	DDir_global	0.289	0.283 ± 0.026 (2.1%)	0.288 ± 0.017 (0.3%)
	H_dir	0.867	0.857 ± 0.035 (1.2%)	0.865 ± 0.023 (0.2%)
	H_rel	3.999	3.926 ± 0.050 (1.8%)	3.964 ± 0.055 (0.9%)
	MDD	2.649	2.646 ± 0.245 (0.1%)	2.657 ± 0.194 (0.3%)
Japanese	DDir_global	0.475	0.476 ± 0.014 (0.2%)	0.475 ± 0.011 (0.0%)
	H_dir	0.998	0.998 ± 0.002 (0.0%)	0.998 ± 0.002 (0.0%)
	H_rel	3.466	3.451 ± 0.051 (0.4%)	3.457 ± 0.034 (0.3%)
	MDD	2.894	2.880 ± 0.162 (0.5%)	2.891 ± 0.095 (0.1%)
Hindi	DDir_global	0.387	0.385 ± 0.009 (0.4%)	0.389 ± 0.008 (0.6%)
	H_dir	0.963	0.961 ± 0.006 (0.1%)	0.964 ± 0.005 (0.1%)
	H_rel	3.706	3.683 ± 0.043 (0.6%)	3.705 ± 0.035 (0.0%)
	MDD	3.305	3.269 ± 0.138 (1.1%)	3.288 ± 0.122 (0.5%)

## Appendix D. Formal Definitions of the 29 Structural Features

For a dependency tree, let *E* be the set of non-root dependency arcs (punctuation excluded). Each arc is a triple (h, d, r), where h is the head token, d the dependent, and r the universal dependency relation. pos(·) denotes linear position in the sentence; |E| is the total number of arcs and |E_r| the number bearing relation r. An arc is head-initial if pos (h) < pos (d), and head-final otherwise.

- Dependency-based features (23)

### Appendix D.1. Direction and Entropy

1. DDir_global—global head-initiality ratio:

DDir_global = (number of head-initial arcs)/|E|

2. H_dir—directional entropy:

H_dir = −p_HI·log_2_ p_HI − p_HF·log_2_ p_HF, where p_HI and p_HF are the proportions of head-initial and head-final arcs (H_dir = 0 for fully fixed ordering, 1 for a 50/50 split).

3. H_rel—dependency-relation distribution entropy:

H_rel = −Σ_i_ p(r_i_)·log_2_ p(r_i_), where p(r_i_) = |E_{r_i_}|/|E| and the sum runs over the N_r attested relation types.

4–21. DDir(r)—relation-specific head-initiality ratio:

DDir(r) = (head-initial arcs with relation r)/|E_r|, computed for each of the following 18 relations: acl, advcl, advmod, amod, case, cc, ccomp, compound, conj, det, discourse, nmod, nsubj, nummod, obj, obl, parataxis, xcomp.

### Appendix D.2. Distance

22. MDD—mean dependency distance:

MDD = (1/|E|) · Σ_{(h,d)∈E} |pos(h) − pos(d)|

23. VarDL—variance of dependency distance:

VarDL = (1/|E|) · Σ_{(h,d)∈E} (|pos(h) − pos(d)| − MDD)^2^

B. Constructional and frequency features (6)

24. CCompRatio—complement-clause ratio: |E_ccomp|/|E|

25. AdvclRatio—adverbial-clause ratio: |E_advcl|/|E|

26. AclRatio—adnominal-clause ratio: |E_acl|/|E|

27. CoordRatio—coordination ratio: |E_conj|/|E|

28. Transitivity—transitivity index: |E_obj|/(number of VERB tokens)

29. CaseRatio—case-marking ratio: |E_case|/|E|

*Note.* All relations were collapsed to the universal core inventory; language-specific subtypes (e.g., nmod:poss, obl:tmod) were merged into their base relation before computation. One additional feature, DDir_balance (= (N_HI − N_HF)/|E|), was extracted initially but removed as an exact linear transform of DDir_global (Section 3.4).

## References

[1] Gray R.D., Jordan F.M. (2000). Language Trees Support the Express-Train Sequence of Austronesian Expansion. Nature.

[2] Gray R.D., Atkinson Q.D. (2003). Language-Tree Divergence Times Support the Anatolian Theory of Indo-European Origin. Nature.

[3] Birchall J., Dunn M., Greenhill S.J. (2016). A Combined Comparative and Phylogenetic Analysis of the Chapacuran Language Family. Int. J. Am. Linguist..

[4] Sagart L., Jacques G., Lai Y., Ryder R.J., Thouzeau V., Greenhill S.J., List J.-M. (2019). Dated Language Phylogenies Shed Light on the Ancestry of Sino-Tibetan. Proc. Natl. Acad. Sci. USA.

[5] Heggarty P., Anderson C., Scarborough M., King B., Bouckaert R., Jocz L., Kümmel M.J., Jügel T., Irslinger B., Pooth R. (2023). Language Trees with Sampled Ancestors Support a Hybrid Model for the Origin of Indo-European Languages. Science.

[6] Kroeber A., Chretien C. (1937). Quantitative Classification of Indo-European Languages. Language.

[7] Sapir E. (1921). How Languages Influence Each Other. Language: An Introduction to the Study of Speech.

[8] Meillet A., Vendryes J. (1938). Introduction à L’étude Comparative Des Langues Indo-Européennes.

[9] Thomason S.G. (2020). Contact Explanations in Linguistics. The Handbook of Language Contact.

[10] Whitney W.D. (1881). The Life and Growth of Language: An Outline of Linguistic Science.

[11] Hartmann F., Walkden G. (2024). The Strength of the Phylogenetic Signal in Syntactic Data. GLOSSA J. Gen. Linguist..

[12] Johanson L., Robbeets M. (2010). Transeurasian Verbal Morphology in a Comparative Perspective: Genealogy, Contact, Chance.

[13] Robbeets M., Bouckaert R., Conte M., Savelyev A., Li T., An D.-I., Shinoda K., Cui Y., Kawashima T., Kim G. (2021). Triangulation Supports Agricultural Spread of the Transeurasian Languages. Nature.

[14] Li W., Liu H. (2026). GDUD: A Reproducible Pipeline for Constructing and Validating Universal Dependencies Treebanks from Grammatical Descriptions, with Application to 15 Low-Resource Tungusic and Mongolic Languages. Trans. Asian Low-Resour. Lang. Inf. Process..

[15] Li W., Liu H. (2026). The Phylogenetic Signal of Syntactic Structure: Limits and Patterns in the Indo-European Language Family. Lang. Dyn. Change.

[16] Witsen N. (1692). Noord En Oost Tartarye.

[17] Ramstedt G.J. (1952). Introduction to Altaic Linguistics.

[18] Miller R.A. (1971). Japanese and the Other Altaic Languages.

[19] Blažek V. (2006). Current Progress in Altaic Etymology. Linguist Online. http://www.phil.muni.cz/linguistica/art/blazek/bla-004.pdf.

[20] Robbeets M. (2007). How the Actional Suffix Chain Connects Japanese to Altaic. Turk. Lang..

[21] Dybo A.V., Starostin G.S. (2008). In Defense of the Comparative Method, or the End of the Vovin Controversy. Asp. Comp. Linguist..

[22] Boller A. (1857). Nachweis, Daß Das Japanische Zum Ural-Altaischen Stamme Gehört. Sitzungsberichte Philos.-Hist. Cl. Kais. Akad. Wiss..

[23] Georg S. (1999). Is Japanese Related to Korean, Tungusic, Mongolic and Turkic?.

[24] Clauson G. (1956). The Case against the Altaic Theory. Cent. Asiat. J..

[25] Doerfer G. (1963–1975). Türkische und Mongolische Elemente im Neupersischen, unter Besonderer Berücksichtigung Älterer Neupersischer Geschichtsquellen, vor Allem der Mongolen- und Timuridenzeit.

[26] Poppe N. (1965). Introduction to Altaic Linguistics.

[27] Miller R.A. (1996). Languages and History: Japanese, Korean and Altaic.

[28] Starostin S., Dybo A., Mudrak O. (2003). Etymological Dictionary of the Altaic Languages.

[29] Georg S. (2005). Reply (to Starostin Response, 2005). Diachronica.

[30] Georg S. (2004). Review of Etymological Dictionary of the Altaic Languages (2003). Diachronica.

[31] Blazek V., Schwarz M. (2014). Jmenná Deklinace v Altajskych Jazycích. Linguist. Brun..

[32] Street J.C. (1962). The Language of the Secret History of the Mongols.

[33] Tekin T. (1994). A Grammar of Orkhon Turkic.

[34] Robbeets M. (2015). Diachrony of Verb Morphology: Japanese and the Transeurasian Languages.

[35] Robbeets M., Bouckaert R. (2018). Bayesian Phylolinguistics Reveals the Internal Structure of the Transeurasian Family. J. Lang. Evol..

[36] Hattori S. (1959). Nihongo No Keitō [The Origins of the Japanese Language].

[37] Kobashi M., Tanaka K. Suuriteki Shuhō o Mochiita Nihongo No Keitō Ni Kansuru Kōsatsu [A Study on the Genealogical Relationships of Japanese Using Mathematical Methods]. Proceedings of the In Gengo Shori Gakkai Dai 17-Kai Nenji Taikai Happyo Ronbunshu [Proceedings of the 17th Annual Meeting of the Association for Natural Language Processing].

[38] Ono S. (1981). Nihongo to Tamirugo [Japanese and Tamil].

[39] Benedict P.K. (1990). Japanese/Austro-Tai.

[40] Matsumoto K. (2007). Sekai Gengo No Naka No Nihongo: Nihongo Keitōron No Arata Na Chihei.

[41] Sakiyama O. (2012). Japanese as a Mixed Language: Sound Law and Semantic Change from Proto-Austronesian to Ancient Japanese. Bull. Natl. Mus. Ethnol..

[42] Robbeets M., Hickey R. (2017). The Transeurasian Languages. The Cambridge Handbook of Areal Linguistics.

[43] Yurayong C., Szeto P.Y. (2020). Altaicization and De-Altaicization of Japonic and Koreanic. Intern. J. Eur. Linguist..

[44] Vovin A. (2005). The End of the Altaic Controversy [Review of Starostin et al. (2003)]. Cent. Asiat. J..

[45] Vovin A. (2017). Koreo-Japonica: A Re-Evaluation of a Common Genetic Origin.

[46] Tian Z., Tao Y., Zhu K., Jacques G., Ryder R.J., Alonso de la Fuente J.A., Antonov A., Xia Z., Zhang Y., Ji X. (2022). Triangulation Fails When Neither Linguistic, Genetic, nor Archaeological Data Support the Transeurasian Narrative. bioRxiv.

[47] Jiang D., Meng W. (2026). From the Altaic Language Family to the Transeurasian Languages: Traditional Genealogical Classification and Algorithmic Clustering. *Dangdai Yuyanxue* [Contemporary Linguistics]. ChinaXiv.

[48] Liu H. (2008). Dependency Distance as a Metric of Language Comprehension Difficulty. J. Cogn. Sci..

[49] Wang Y., Liu H. (2017). The Effects of Genre on Dependency Distance and Dependency Direction. Lang. Sci..

[50] Williamson D.M., Xi X., Breyer F.J. (2012). A Framework for Evaluation and Use of Automated Scoring. Educ. Meas..

[51] de Marneffe M.-C., Dozat T., Silveira N., Haverinen K., Ginter F., Nivre J., Manning C.D. (2014). Universal Stanford Dependencies: A Cross-Linguistic Typology. Proceedings of the Ninth International Conference on Language Resources and Evaluation (LREC’14).

[52] Jiang J., Liu H. (2015). The Effects of Sentence Length on Dependency Distance, Dependency Direction and the Implications–Based on a Parallel English–Chinese Dependency Treebank. Lang. Sci..

[53] Tesnière L. (1959). Éléments de Syntaxe Structurale.

[54] Mel’čuk I., Agel V., Eichinger L.M., Eroms H.W., Hellwig P., Heringer H.J., Lobin H. (2003). Levels of Dependency in Linguistic Description: Concepts and Problems. Dependency and Valency: An International Handbook of Contemporary Research.

[55] Hudson R. (2007). Language Networks: The New Word Grammar.

[56] Liu H. (2010). Dependency Direction as a Means of Word-Order Typology: A Method Based on Dependency Treebanks. Lingua.

[57] Niu R., Wang Y., Liu H. (2023). The Cross-Linguistic Variations in Dependency Distance Minimization and Its Potential Explanations. Proceedings of the PACLIC 37.

[58] Greenberg J.H., Greenberg J. (1963). Some Universals of Grammar with Particular Reference to the Order of Meaningful Elements. Universals of Language.

[59] Dryer M.S. (1992). The Greenbergian Word Order Correlations. Language.

[60] Dryer M.S. (1997). On the 6-Way Word Order Typology. Stud. Lang..

[61] Revell L.J. (2012). Phytools: An R Package for Phylogenetic Comparative Biology (and Other Things). Methods Ecol. Evol..

[62] Lewis P.O. (2001). A Likelihood Approach to Estimating Phylogeny from Discrete Morphological Character Data. Syst. Biol..

[63] Ronquist F., Teslenko M., Van Der Mark P., Ayres D.L., Darling A., Höhna S., Larget B., Liu L., Suchard M.A., Huelsenbeck J.P. (2012). MrBayes 3.2: Efficient Bayesian Phylogenetic Inference and Model Choice Across a Large Model Space. Syst. Biol..

[64] Saitou N., Nei M. (1987). The Neighbor-Joining Method: A New Method for Reconstructing Phylogenetic Trees. Mol. Biol. Evol..

[65] d’Huy J. (2025). Quand l’arbre ne tient plus qu’à ses branches: Trois essais récents pour reconstruire un arbre mondial des langues. Humanit. Numériques.

